# Development of Mycoinsecticides: Advances in Formulation, Regulatory Challenges and Market Trends for Entomopathogenic Fungi

**DOI:** 10.3390/jof12010007

**Published:** 2025-12-22

**Authors:** Joel C. Couceiro, Martyn J. Wood, Andronikos Papadopoulos, Juan J. Silva, John Vontas, George Dimopoulos

**Affiliations:** 1 Institute of Molecular Biology and Biotechnology, Foundation for Research and Technology Hellas (IMBB-FORTH), Nikolaou Plastira 100, GR-70013 Heraklion, Greece; joel_couceiro@imbb.forth.gr (J.C.C.); martyn_wood@imbb.forth.gr (M.J.W.); andron_papadopoulos@imbb.forth.gr (A.P.); juan_silva@imbb.forth.gr (J.J.S.); vontas@imbb.forth.gr (J.V.); 2 Pesticide Science Laboratory, Department of Crop Science, Agricultural University of Athens, Iera Odos 75, Votanikos, GR-11855 Athens, Greece; 3 W. Harry Feinstone Department of Molecular Microbiology and Immunology, Bloomberg School of Public Health, Johns Hopkins University, 615 N. Wolfe Street, Baltimore, MD 21205, USA

**Keywords:** entomopathogenic fungi, formulation, mycoinsecticides, microbial control

## Abstract

Bioinsecticides offer eco-friendly alternatives to chemical insecticides and thereby meet the need for sustainable pest control. Entomopathogenic fungi (EPF) represent one of the core classes of microbial insecticides, distinguished by their advantageous contact-based mode of action. Several products have been successfully commercialized, and with continuing improvements to the technology, the market size for EPF continues to grow. The translation of EPF into reliable field performers relies upon formulation technologies that ensure product quality, stability, virulence, and cost-effectiveness. Current formulations comprise diverse solid and liquid states (e.g., wettable powders, oil dispersions) that deliver a range of propagules (conidia, blastospores, microsclerotia). While advanced approaches like nanoparticle encapsulation show promise, some limitations hinder their widespread use. Major constraints include maintaining fungal viability during storage/transport and protecting propagules from harsh environmental factors post-application. Regulatory requirements also present significant barriers to widespread uptake. Addressing these formulation challenges through continued research is essential for advancing mycoinsecticide technology and increasing their contribution to integrated pest management. This review aims to present the latest scientific advances in EPF formulation technologies and application strategies, alongside an overview of current regulatory frameworks and an up-to-date analysis of registered microbial biopesticide products in some of the world’s largest markets.

## 1. Introduction

Entomopathogenic fungi (EPF) represent a large group of microorganisms that are natural antagonists of arthropods by infecting, parasitizing, and killing a very broad range of insect species [1,2,3]. While historical accounts of insect disease trace back over two millennia to Aristotle, the definitive demonstration of a microorganism causing insect disease occurred in 1835. Agostino Bassi, widely regarded as the “Father of Insect Pathology”, documented for the first time the transmission of a fungal pathogen, now identified as *Beauveria bassiana* (Bals.-Criv.) Vuill., between caterpillars of the silkworm, *Bombyx mori* L. [4,5]. Several decades later, in 1879, the Russian scientist Elie Metchnikoff isolated the fungus *Metarhizium anisopliae* (Metschn.) Sorokin (initially reported as *Entomophthora anisopliae*) from the wheat cockchafer and notably proposed its utilization for the control of soil-borne insect pests [5,6,7].

The earliest documented mass-production of a microbial entomopathogen is attributed to Krassilstchik; during the summer of 1884 in Smila (present-day Ukrainian territory), he managed a small facility that yielded 55 kg of pure *M. anisopliae* spores (reported as *Isaria destructor*) aimed at controlling the sugar beet weevil, *Asproparthenis punctiventris* (Germar) (reported as *Cleonus punctiventris*) [8]. Interest in harnessing fungi as microbial control agents persisted through the late 19th and early 20th centuries. This period saw the initiation of several international projects applying EPF species across different agricultural systems to combat pest insects. These efforts produced variable outcomes, reporting both successful control instances and numerous failures, the latter primarily attributed to the absence of optimal climatic conditions required for establishing epizootics (for detailed accounts, refer to [9]. In the decades after World War II, the emergence of synthetic chemical insecticides resulted in diminished research attention towards EPF [10]. However, interest dramatically resurged during the 1970s and 1980s, following the publication of ‘Silent Spring’ in 1962 and a general increase in scientific and popular understanding of the detrimental environmental impacts associated with chemical insecticides [11].

Within the Kingdom Fungi, the Order Hypocreales (phylum Ascomycota) contains the most well-known EPF. Although entomopathogenicity is present in other groups, such as Entomophthorales (phylum Zoopagomycota), in this review, we focus only on hypocrealean EPF, due to their higher relevance and broad usage within the field. Numerous genera of EPF have been rigorously investigated for their potential as biological control agents, due to their broad host range, low environmental impact, and ability to be formulated to target specific hosts. A key advantage is their capacity to initiate infection directly through the insect cuticle (contrasting with bacteria and viruses that often require ingestion), enabling them to also effectively target sucking insects, such as aphids and whiteflies [12].

**Figure 1:**
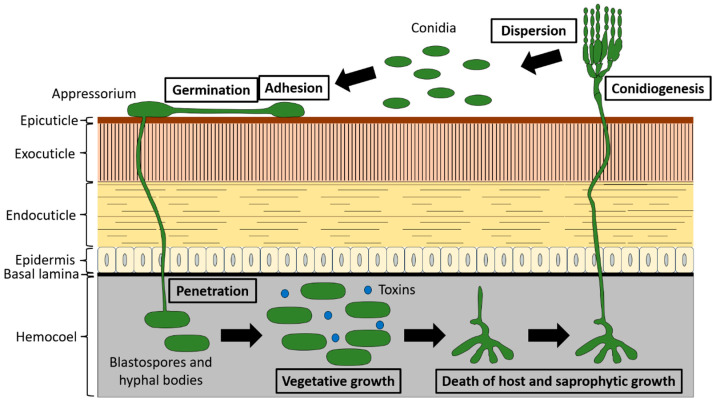
General infection cycle of a hypocrealean entomopathogenic fungus. The cycle begins with conidial attachment to the host cuticle via hydrophobic interactions. Under favorable conditions (e.g., temperature, humidity, nutrient availability), conidia germinate, potentially forming specialized structures (e.g., appressoria) to breach the cuticle. The fungus then penetrates the host using mechanical pressure and enzymatic degradation (e.g., proteases, chitinases, lipases), reaching the hemocoel. Within the host, the fungus differentiates into hyphal bodies and blastospores, consuming nutrients in the hemolymph, colonizing tissues through vegetative growth, and producing insecticidal, immunosuppressive, and antibiotic secondary metabolites, ultimately causing host death. Under suitable conditions, the fungus emerges from the cadaver, forms conidiophores and produces new conidia, which are dispersed (via wind, rain, animal vectors) and can infect new hosts. Adapted from Mascarin and Jaronski [23].

The traditional infection cycle of a hypocrealean EPF is shown in Figure 1. The process commences when aerial conidia establish contact with a suitable host. This leads to the adhesion of propagules to the cuticle, a process facilitated largely through hydrophobic interactions mediated predominantly by hydrophobins [13,14]. In *Metarhizium* spp., the adhesin-like protein Mad1 is reported to mediate adhesion to the host surface [15], while the protein Adh2 plays this role in *B. bassiana* [16]. Following adhesion, contingent upon favorable environmental and physiological conditions (e.g., adequate temperature and humidity, sufficient nutritional cues), conidia germinate and may differentiate specialized structures, such as appressoria, to facilitate penetration into the host [17,18]. During this stage, the fungus secretes a diverse array of enzymes, principally proteases, chitinases, and lipases. Through a combination of mechanical pressure and enzymatic degradation, the fungus breaches the cuticle to gain access to the insect’s hemocoel [18,19]. Once inside the host, the fungus proliferates into hyphal bodies and blastospores, yeast-like structures that actively consume hemolymph nutrients, colonize host tissues via vegetative growth, and produce a variety of secondary metabolites exhibiting insecticidal, immunosuppressive, and antibiotic properties [20,21,22]. Following the death of the insect, under conducive conditions, the pathogen transitions to saprophytic growth, emerging from the cadaver and forming conidiophores on the external surface to produce new aerial conidia, thereby completing its life cycle [2,21].

Over the past few decades, mounting concerns about the toxicological effects of chemical insecticides on the environment and on human health, coupled with the increasing prevalence of insecticide resistance among major crop pests, have significantly motivated research into the development of environmentally safe alternatives for pest management [12,24,25]. Reflecting this trend, the European Union (EU) introduced the Sustainable Use of Pesticides Directive (Directive 2009/128/EC) in 2009, aiming to mitigate the impacts of chemical insecticides while promoting integrated pest management (IPM) and non-chemical alternatives for plant protection [26]. Countries including Brazil, China, and the United States have similarly established regulatory bodies and/or regulations to manage or restrict chemical insecticide usage, while stimulating the adoption of biological control methods and the development of bioinsecticides, often accompanied by measures to facilitate the registration process for these novel products [24].

Despite increasing interest and regulatory support, the development, commercialization and application of mycoinsecticides continue to encounter obstacles that impede their widespread adoption. These include the detrimental effects of abiotic factors on fungal propagule viability and infectivity, field efficacy variability and production cost challenges. To mitigate these limitations, researchers and commercial entities commonly employ formulation techniques to optimize the bioinsecticide action of these microorganisms [24,27,28]. In this review, we provide a comprehensive overview of the most frequently utilized formulation types for mycoinsecticides and further discuss emergent formulation technologies showing significant potential for near-future commercial exploration. Then, we examine the current state of the mycopesticide market, as well as the regulation process in four key markets (USA, Brazil, China, and the EU). Finally, we address current challenges to the successful implementation and integration of advanced fungal formulations within IPM programs.

## 2. Traditional Fungal Formulations

**Figure 2:**
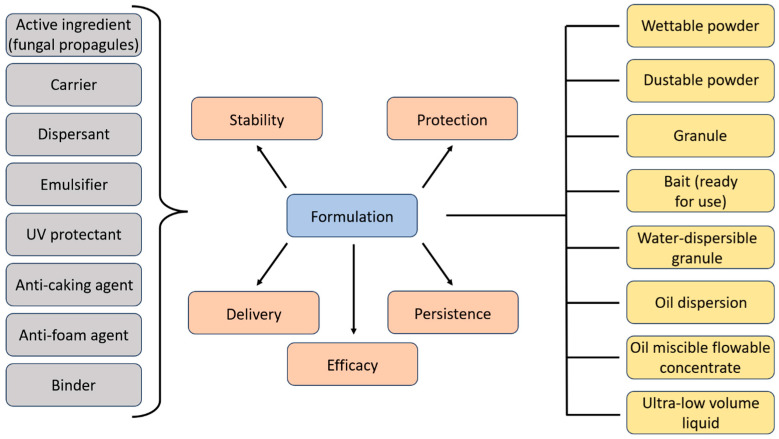
Several types of ingredients (gray boxes) can be incorporated into formulations, providing different benefits (pink boxes). The most common types of formulations of entomopathogenic fungi are shown in the yellow boxes.

Within the domain of microbial control of insects, formulation involves the process of incorporating specific compounds with a selected microorganism to optimize its performance. The primary objectives are to enhance the agent’s stability, provide protection against deleterious environmental factors [such as ultraviolet (UV) radiation or desiccation], and improve delivery mechanisms that are compatible with the target environment and the overall efficacy against the pest organism [27,29]. This enhancement is typically achieved through the informed use of additives, non-insecticidal ingredients selected for specific functions and that crucially must not compromise the viability or virulence of the mycoinsecticide product; examples include humectants to retain moisture, sunscreens for UV protection, and dispersants to ensure even distribution [30] (Figure 2).

The practice of formulation EPF is well established, with numerous species having been successfully developed into commercial mycoinsecticides. A review by Faria and Wright [31] highlights a variety of such EPF species, including *B. bassiana*, *Beauveria brongniartii* (Sacc.) Petch, *M. anisopliae*, *Metarhizium acridum* (Driver & Milner) J.F. Bisch, S.A. Rehner & Humber (formerly *M. anisopliae* var. *acridum*), *Metarhizium rileyi* (Farl.) Kepler, S.A. Rehner & Humber (formerly *Nomuraea rileyi*), *Cordyceps fumosorosea* (Wize) Kepler, B. Shrestha & Spatafora (formerly *Isaria fumosorosea*), *Hirsutella thompsonii* F.E. Fisher, and *Akanthomyces dipterigenus* (Petch) Spatafora, Kepler, Zare & B. Shrestha (formerly *Lecanicillium longisporum*).

The successful development of any microbial formulation necessitates careful consideration of numerous interconnected factors. These encompass the intrinsic biological properties of the microbial agent, the specific biology and behavioral patterns of the target host, the physicochemical characteristics of the formulation components, the environmental conditions prevalent during application (including natural factors potentially affecting pathogen survival and infectivity), and the practical needs and expectations of the end-user; an extensive exploration of the significance of these factors is reviewed by Leggett et al. [32].

Building upon these foundational concepts, the subsequent sections of this review will present a detailed examination of the most traditional and relevant EPF-based bioinsecticide formulation types. This discussion incorporates technical concentrates, which, despite not being a formulation, hold significant relevance as starting materials for mycoinsecticides. Additionally, suspension concentrates will be addressed; a formulation type less frequently employed for EPF but notably subject to misidentification. Representative examples of mycoinsecticides that have undergone development and are currently marketed, illustrating diverse formulation approaches, are cataloged in Table 1. A summary of the formulation types, with their main advantages and disadvantages, is presented in Table 2.

### 2.1. Technical Concentrate (TK)

While technical concentrates (TKs) are not considered a formulation like those presented in the subsequent sections, we deemed their consideration within this review essential due to their significant practical application. Specifically, in numerous countries, these concentrates are directly utilized as end-products in field settings for pest control [31].

CropLife International provides a definition characterizing TKs as products containing the active ingredient (a.i.) along with its associated impurities; they may optionally contain minimal amounts of additives and diluents if deemed necessary [33]. Offering a complementary perspective tailored to biological agents, the FAO/WHO Joint Meeting on Pesticide Management defines microbial TKs as comprising the microorganism itself, its associated metabolites produced during the growth phase, and residual materials originating from the growth medium, all without intentional alteration following production [29]. Accordingly, products classified under this category often consist of fungus-colonized solid substrates. Such substrates are commonly generated through solid-state or biphasic fermentation techniques, typically employing cereal grains or similar matrices for fungal propagation.

### 2.2. Solid Formulations

#### 2.2.1. Wettable Powder (WP)

Wettable powder (WP) formulations comprise finely ground powders designed for suspension in water immediately prior to application. A typical WP composition includes 50–80% technical powder (containing the a.i.), 15–45% inert and hydrophilic filler, 1–10% dispersant, and 3–5% surfactant, calculated by weight [27]. The incorporated non-a.i. additives serve critical functions: they stabilize the product to ensure adequate shelf-life, facilitate easy miscibility when mixed with water for spraying, and provide protection against environmental stressors that could degrade the active ingredient. For example, silica may be included as a filler and anti-caking agent (though amounts might be limited due to potential abrasion of equipment); dispersants are essential for maintaining the a.i. particles in suspension within the water carrier; surfactants lower the surface tension at the liquid-solid interface to improve wetting and coverage; and UV protectants can be added to function as photostabilizers, shielding the fungal propagules from damaging radiation [27,34].

WPs are generally employed in foliar spray applications using standard sprayers, making them especially suitable for agricultural crop foliage pests. The field performance of EPF WP formulations against agricultural pests is well-documented through numerous trials. Representative studies include a WP formulation of *B. bassiana* conidia, which, when sprayed at concentrations of 3.75–6.25 × 10^12^ conidia/ha, significantly reduced nymph populations of the whitefly *Pealius mori* Takahashi on mulberry plants, demonstrating efficacy comparable to the chemical insecticide buprofezin [35]. Similarly, a WP formulation of *Akanthomyces lecanii* (Zimm.) Spatafora, Kepler & B. Shrestha (reported as *Lecanicillium lecanii*), applied at 1500 g/ha, substantially decreased populations of the tea thrips *Scirtothrips bispinosus* (Bagnall) in field trials [36]. A WP formulation of *C. fumosorosea* was reported to control the bagworm *Metisa plana* Walker as effectively as the synthetic insecticide flubendiamide, without causing detrimental side effects to the beneficial oil palm pollinator, *Elaeidobius kamerunicus* Faust [37]. In field trials against the Asian citrus psyllid, *Diaphorina citri* Kuwayama, a major pest of citrus and vector of bacteria that cause the disease Huanglongbing, a WP formulation of *Purpureocillium lilacinum* (Thom) Luangsa-ard, Houbraken, Hywel-Jones & Samson (formerly *Paecilomyces lilacinum*) resulted in higher psyllid mortality than an unformulated conidial suspension, and although the WP acted slower in comparison to chemical insecticides, it had greater persistence as a long-term control agent [38]. Furthermore, research utilizing blastospores to prepare WPs of *Metarhizium robertsii* J.F. Bisch., S.A. Rehner & Humber found that these formulations achieved control of the corn leafhopper *Dalbulus maidis* (DeLong & Wolcott) comparable to that of fresh, unformulated blastospores [39].

While WPs are primarily employed in agriculture, they have occasionally also been used against insects of medical and veterinary importance. For instance, a WP of *Metarhizium brunneum* Petch, alone or in combination with low doses of the insecticide deltamethrin, proved successful in controlling house flies (*Musca domestica* L.), a pest that can transmit diseases and negatively impact the growth of livestock and poultry [40].

Several advantages are associated with WP formulations: they are generally easy to handle, compatible with conventional spray application technologies, can exhibit prolonged shelf-life (potentially exceeding 18 months, provided moisture content is rigorously controlled), and typically demonstrate low phytotoxicity [41,42]. However, certain drawbacks exist, including the potential for dust generation during manufacturing and handling (posing inhalation risks), the possibility of clogging spray equipment, and the rapid settling of particles in the spray tank, which usually necessitates continuous agitation to maintain a uniform suspension [34,43]. Notwithstanding these limitations, WP formulations represent one of the most prevalent categories among commercial mycoinsecticides [31,44].

#### 2.2.2. Dustable Powder (DP)

Dustable powder (DP) formulations consist of finely ground powders characterized by small particle sizes, typically within the 50–100 μm range. These products are composed of the a.i. blended with inert fillers and carriers, where the a.i. usually constitutes a relatively low proportion, often less than 10% by weight [27]. Various functional components may be included to optimize the formulation, such as stickers designed to enhance the adhesion of particles onto target surfaces, desiccants acting as anti-caking agents, and stabilizers intended to improve product shelf life [34,43].

The efficacy of EPF in DP formulations has been investigated in several studies. For example, a dust formulation of *M. anisopliae*, when applied to the surfaces of glass jars using varying ratios of conidia to carrier, effectively induced high mortality in the brown-banded cockroach, *Supella longipalpa* (Fabricius) [45]. Likewise, DP formulations of *B. bassiana* and *M. anisopliae* conidia significantly augmented insecticidal efficacy against rice weevils [*Sitophilus oryzae* (L.)], lesser grain borers [*Rhyzopertha dominica* (Fabricius)], and red flour beetles [*Tribolium castaneum* (Herbst)], three major stored-grain pests, in comparison to treatments using unformulated conidia [46]. Similarly, DP formulations of *B. bassiana* with diatomaceous earth increased mortality and decreased progeny production of *R. dominica* in wheat [47]. These examples demonstrate how DP formulations are mainly used for surface treatments for the management of pests of stored products, where direct contact with insects is expected. While mainly of agricultural relevance, their principle can also extend to veterinary contexts, such as poultry houses infested with cockroaches or mites. For instance, a dust formulation of *B. bassiana* mixed with diatomaceous earth significantly increased mortality of the poultry red mite, *Dermanyssus gallinae* De Geer [48], while a similar *M. anisopliae*-based dust enhanced the mortality of *Triatoma infestans* (Klug), the vector of Chagas disease, particularly under low relative humidity [49].

A key characteristic of DPs is that they are normally supplied as ready-to-use, allowing for direct application without the requirement for dilution or suspension in water. However, a notable consideration pertains to potential user safety concerns. Because of their very fine particle size and propensity for easy aerial dispersal, dusts can present inhalation hazards and potentially trigger allergenic responses. Consequently, their application necessitates adherence to precautionary measures that minimize exposure risks.

#### 2.2.3. Granule (GR)

Granule (GR) formulations are defined as dry, discrete particles, typically sized between 5–10 mm^3^, which generally incorporate the a.i., a binder and a carrier material. The concentration of the a.i. in these formulations commonly ranges from 5 to 20% [27,41]. A prevalent manufacturing process involves blending the components into a paste, which is subsequently extruded or passed through a sieve to generate granules of the desired dimensions, followed by a drying step if necessary. Significant advantages of GR formulations, particularly when compared to powder formulations like WPs and DPs, include their non-caking properties and substantially reduced risk of inhalation exposure to the user. Furthermore, GRs constitute a more elaborate formulation strategy than TKs, being characterized by a greater number of components, production to a uniform size specification, and typically ensuring strong adhesion of the fungal conidia or other propagules to the substrate matrix.

This formulation type is particularly suitable for targeting pests residing in the soil. Following dispersal onto or into the soil, the granules absorb available moisture; this hydration allows the EPF to germinate and subsequently colonize the immediate soil environment, a process that emulates the natural release of spores from an infected insect cadaver. The strategic addition of nutrients into the granule formulation can further stimulate fungal growth and enhance sporulation directly from the granule itself [44,50]. Consequently, due to this localized proliferation and release mechanism, EPF delivered via granules can demonstrate extended persistence (residual activity) within the soil [51], thereby offering potential for long-term pest control.

The efficacy of EPF delivered as granules has been validated in various studies involving agricultural pests. For example, formulations containing microsclerotia of *M. anisopliae* and *M. brunneum* (isolate F52, which was reported at the time as *M. anisopliae*), prepared using diatomaceous earth, achieved 90–100% mortality against the sugar beet root maggot, *Tetanops myopaeformis* (Röder) [52]. Similarly, microsclerotia from the same *M. brunneum* isolate (F52), when formulated into clay-based granules, were demonstrated to be effective against larvae of the lesser mealworm, *Alphitobius diaperinus* (Panzer) [53].

In addition to crop protection, their adaptation to dissemination devices demonstrates the potential of GR formulations in medical entomology, where they can target mosquito vectors breeding in or near soil and water habitats, and in veterinary contexts for managing tick populations in pastures. For example, granular *M*. *robertsii* microsclerotia and blastospore formulations, developed using microcrystalline cellulose and Psyllium, successfully reduced larval populations of the cattle tick *Rhipicephalus microplus* (Canestrini) in semi-field experiments [51]. Under laboratory conditions, a prototype granular formulation of *M. brunneum* microsclerotia increased mortality of both fed and unfed nymphs of the black-legged tick, *Ixodes scapularis* Say, an important vector of several diseases, including Lyme disease [54]. Granular formulations of *Metarhizium humberi* Luz, Rocha & Delalibera, containing conidia, microsclerotia or both, were applied to polyethylene terephthalate carpets placed inside dissemination devices designed to attract adult females of *Aedes aegypti* (L.). Laboratory and semi-field assays showed increased mosquito mortality over the course of 20 days, while field trials resulted in a lower number of eggs in ovitraps during a period of 16-week intervention period within a 24-week trial [55]. Additionally, the GR formulation of *M. brunneum* microsclerotia applied to potting mix caused premature eclosion of *A. aegypti* eggs in laboratory studies, leading to lower larval survival and lower adult emergence [56], and granules of *M. humberi* microsclerotia showed high virulence against *A. aegypti* adults [57].

Despite their placement within the soil profile, where some degree of shielding might be provided by the overlying plant canopy or soil particles, exposure to UV radiation can still present a significant challenge to the viability of EPF in granules located at, or near, the soil surface. Therefore, the inclusion of effective UV protectants is often a critical consideration during the development and optimization of durable GR formulations [41].

#### 2.2.4. Bait (Ready for Use) (RB)

Bait formulations are engineered for ingestion by the target pest. This is achieved through the incorporation of attractants designed to lure insects to consume the bait, rendering them particularly useful against insect species that actively forage or feed at the soil surface [29,44].

Several studies have explored the development and efficacy of baits incorporating EPF. For instance, *B. bassiana*, *C. fumosorosea* (reported as *I. fumosorosea*), and *P. lilacinum* were formulated into baits using citric pulp as an attractant and presented to colonies of the leaf-cutting ant *Atta sexdens rubropilosa* Forel. While these baits did not result in complete colony elimination, they were readily accepted and transported into the nest (90–100% of granules carried) and led to observable effects such as increased waste volume and a higher number of dead ants accumulating in the colony’s waste piles [58]. In another study, baits containing *M. anisopliae* combined with sugarcane molasses proved highly effective against termites (*Microcerotermes diversus* Silvestri), inducing substantial mortality at the tested conidial concentrations of 3.7 × 10^7^ and 3.5 × 10^8^ conidia/mL [59].

In non-agricultural contexts, *M. anisopliae* has also been successfully formulated as baits aimed at controlling house flies (*M. domestica*). Renn et al. [60] developed a bait consisting of 20% sucrose solution supplemented with 0.2% methyl hydroxybenzoate and 0.1% streptomycin sulfate to suppress contaminants, combined with the fly attractant z-9-tricosene, and overlaid with an *M. anisopliae* culture; this formulation achieved mortality rates of 95–100% after ten days. Baker et al. [61] tested a granular bait of *M. anisopliae*-coated white rice grains, with skim milk powder as an additive, and observed fly mortality of over 99% after seven days. Although attractive toxic sugar baits (ATSBs) are increasingly investigated for mosquito control, using mainly chemical insecticides [62,63], no studies have reported the incorporation of EPF into ATSB systems.

#### 2.2.5. Water-Dispersible Granule (WG)

Water-dispersible granule (WG) formulations consist of granules meticulously engineered to disintegrate rapidly upon mixing with water, thereby creating a homogeneous suspension amenable to application through conventional spray systems [29]. This formulation type was developed, in part, to offer enhanced end-user safety compared to traditional powder formulations, significantly mitigating the risks associated with dust inhalation during handling and preparation [64,65]. Typically, the concentration of the a.i. in WGs is substantial, falling within the range of 50–90% [66]. Similarly to WPs, WG formulations incorporate both wetting and dispersing agents; however, the proportion of dispersant is commonly higher in WGs to facilitate swift and thorough dispersion upon dilution [41]. Other frequent additives are anti-caking agents to ensure good flowability of the granules, and potentially a specific disintegrating agent designed to accelerate the breakdown of granules within the spray tank [67]. The production of WGs can be achieved through various established granulation techniques, including pan granulation, high-speed mixing agglomeration, extrusion granulation, fluid bed spray granulation, and spray drying [66]. Compared to liquid formulations, WGs present advantages regarding ease of storage and transport [68] and are recognized for possessing good storage stability characteristics, thereby prolonging shelf life [69].

While the utility of EPF WG formulations has been investigated, R&D appears relatively limited as of early 2025, regardless of targeting pests of agricultural or medical relevance. One study assessed a *B. bassiana* WG formulation targeting the coffee berry borer, *Hypothenemus hampei* (Ferrari). The experiment showed that mortality levels induced by the WG did not significantly differ from those caused by unformulated conidia. However, the study also observed a tendency towards lower field efficacy and measured an approximate 20% decrease in conidial germination after the formulation process [70]. Such findings, combined with the overall scarcity of published studies focused specifically on EPF WGs, highlight a clear need for additional research to thoroughly explore, evaluate and optimize this formulation approach for effective application as mycoinsecticide products.

### 2.3. Liquid Formulations

#### 2.3.1. Oil Dispersion (OD)

Oil dispersion (OD) formulations are characterized by the dispersion of the a.i., along with required additives (such as carriers, dispersants, and rheological agents), within a water-immiscible fluid phase, which is most commonly an oil. This process yields a stable suspension concentrate intended for dilution with water immediately before application [29]. To facilitate the mixing of this oil-based concentrate into water, the inclusion of appropriate emulsifiers is required. Given the potential for solid a.i. particles like fungal conidia to settle within the oil carrier over time, dispersants may be incorporated to enhance particle compatibility with the liquid medium [71], or alternatively, settled particles can often be effectively resuspended by simple manual agitation prior to dilution [31].

OD presents a particularly advantageous approach for formulating an a.i. that exhibits sensitivity to water. Premature exposure of EPF to water can trigger the spore germination and elevate cell metabolism, consequently rendering the propagules more vulnerable to detrimental environmental factors [72]. Foliar applications of OD formulations often demonstrate superior wetting and spreading properties on target surfaces and can enhance the bioavailability of the a.i. due to the significantly slower evaporation rate of the oil carrier compared to water [41,71]. Extensive research corroborates multiple benefits conferred by OD formulations for EPF, including ease of handling and use, protection of fungal propagules against imbibitional damage [73] and thermal stress [74], increased tolerance to UV exposure [75,76], compatibility with certain chemical fungicides [77], and improved persistence on treated surfaces due to enhanced rainfastness [78]. Furthermore, several studies have reported heightened virulence against target pests, such as beetles [79], psyllids [80], ticks [76,81,82,83], and mosquitoes [84,85,86].

It is important to address terminology accurately, as OD formulations are occasionally misidentified as suspension concentrates (SC, which are discussed below) or emulsifiable concentrates (EC). The term EC is specifically reserved for formulations wherein the active ingredient is dissolved in a solvent system to form a homogeneous liquid concentrate [29], making this designation inappropriate for the inherently particulate nature of EPF suspensions.

#### 2.3.2. Suspension Concentrate (SC)

A noteworthy pattern observed within the scientific literature concerning EPF is the relatively common reference to “suspension concentrate” formulations. However, upon closer scrutiny, many such studies describe formulations where the fungal propagules are dispersed in an oil-based carrier, intended for later dilution in water, a description that defines an OD. Despite suspension concentrates (SCs) appearing to be less frequently utilized for EPF compared to other types, their inclusion in this review is appropriate. The primary reason is to address the widespread confusion between SC and OD terminology and abbreviations, thereby aiding readers in discerning whether a formulation reported in publications genuinely constitutes an SC or is, in fact, an OD. For the purpose of summarizing commercial products (Table 1), items labeled as SC by their manufacturers were maintained under this designation if further details regarding their precise composition (specifically, the nature of the liquid carrier) could not be ascertained from publicly available information provided in product labels or safety data sheets.

Suspension concentrate formulations are defined as a suspension of the a.i. within an aqueous, water-based continuous phase; these concentrates are designed to be diluted further with water before application [29]. The typical composition of a SC includes approximately 20–50% a.i., 2–5% wetting and/or dispersing agents, 5–10% antifreeze agent (to prevent particle aggregation caused by freezing temperatures during storage or transport), and 0.2–2% anti-settling agent (to minimize sedimentation), with water making up the remainder to 100% [66]. Because the active ingredient exists as suspended solid particles rather than being dissolved, agitation of the diluted suspension is necessary prior to and often during application to ensure uniformity [69]. Stickers may optionally be added to enhance the adherence of the applied formulation. Generally recognized advantages of SCs include their non-dusty character, ease of handling and measurement, and favorable safety attributes for both users and the environment.

#### 2.3.3. Oil Miscible Flowable Concentrate (OF)

Oil miscible flowable concentrate (OF) formulations, which are also termed oil miscible suspensions, represent stable dispersions of the a.i. developed specifically for dilution within an organic liquid carrier prior to application. This contrasts distinctly with ODs, which are designed for dilution in water [29]. A direct consequence of this design is that OF formulations do not necessitate the inclusion of emulsifiers, as they are not intended for mixing with an aqueous phase [87]. These properties make OF formulations especially well-suited for deployment using ultra-low volume (ULV) application equipment.

OF formulations are highly relevant for locust and grasshopper control. For example, field trials employing *M. acridum* (identified in the study as *M. anisopliae* var. *acridum*) OF achieved approximately 88% mortality against the grasshopper *Rhammatocerus schistocercoides* (Rehn) 14 days after application [88]. Similarly, field applications of an *M. acridum* OF in Mauritania, Tanzania, and Senegal resulted in a significant reduction in grasshopper and locust populations [89].

#### 2.3.4. Ultra-Low Volume Liquids (UL)

Within the specific context of EPF applications, ultra-low volume liquid (UL) formulations are defined as non-aqueous suspensions featuring the a.i. at a notably high concentration. A key characteristic of UL formulations is that they are supplied ready for application using specialized ultra-low volume (ULV) equipment, without requiring any dilution with water prior to use [29,69]. The application of UL formulations can be performed utilizing either aerial or ground-based ULV equipment, both of which are engineered to atomize the liquid into an extremely fine spray [90].

## 3. Innovations in Formulation of EPF

The effective development of formulations for EPF necessitates careful consideration of multiple critical challenges. Key among these are: ensuring the protection of the a.i. from rapid environmental degradation (e.g., UV radiation, desiccation), extending product shelf life under various storage conditions, guaranteeing compatibility with existing application equipment, controlling the release kinetics of the active agent, managing overall production costs, ensuring occupational safety during manufacture and end-use, and optimizing logistics related to transportation and storage. Modern formulation science endeavors to address these multifaceted requirements, striving to create mycoinsecticide products that exhibit enhanced persistence, improved biological efficacy, potentially offer controlled or targeted release mechanisms, while remaining economically competitive. Current trends indicate a gradual shift away from traditional powdery formulations, such as WPs and DPs, largely driven by concerns regarding inhalation hazards for users. Concurrently, there is burgeoning research interest and activity focused on novel formulation strategies employing advanced materials like nanoparticles and biopolymers.

**Figure 3:**
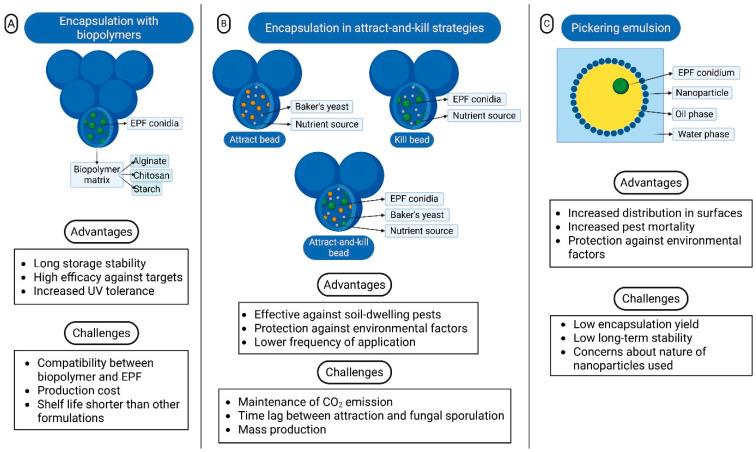
Innovative formulation strategies for entomopathogenic fungi (EPF). (**A**) Biopolymer encapsulation commonly utilizes alginate, chitosan or starch, although other biopolymers can also be employed (Section 3.1). (**B**) Encapsulation for attract-and-kill strategies, where components may be co-encapsulated or separately encapsulated into “attract” (e.g., baker’s yeast and nutrients) and “kill” (EPF and nutrients) beads for co-application. (**C**) Oil-in-water Pickering emulsion, with a capsule containing a single conidium.

Building on this context, the subsequent section of this review provides an overview and discussion of current advances in formulation technology relevant to EPF. Emphasis will be placed on those innovative approaches demonstrating significant potential for the development of next-generation mycoinsecticides. Figure 3 illustrates these innovative formulations and presents their key advantages and challenges they face.

### 3.1. Encapsulation Using Biopolymers

Encapsulation represents an advanced formulation technique where the EPF propagules (a.i.) are encased within a matrix material, frequently polymer-based. This coating acts as a physical barrier, isolating the fungus from deleterious environmental influences such as UV radiation, adverse temperature fluctuations, desiccation, and competition from other microbes. Consequently, encapsulation can lead to a more stable product during storage and post-application, potentially resulting in extended shelf life and residual activity [91,92,93,94]. Moreover, the properties of the encapsulating matrix can be tailored to modulate the release kinetics of the a.i. This allows for the possibility of a slower controlled release, which may enhance the establishment and environmental persistence of the EPF after application, thereby potentially reducing the required dose or frequency of treatments [93,95].

An important consideration during the development of encapsulated EPF is ensuring the biocompatibility of the formulation components with the microorganism itself, guaranteeing that fungal viability and virulence remain unaltered. Several studies have highlighted potential incompatibilities; for instance, Rodrigues et al. [96] reported moderate toxicity when exposing *B. bassiana* to 3% DMSO or 1.5% maltodextrin, and *M. anisopliae* to 2% or 3% DMSO. Likewise, Felizatti et al. [97] observed that lignin (at 1% and 2% *w*/*v*) and humic acid (at 2% *w*/*v*) exhibited toxicity towards *B. bassiana*, which the authors suggested might relate to pH changes induced by these agents in the culture medium. The detrimental effects observed in both studies typically included lower rates of sporulation, germination, and vegetative growth.

Notwithstanding these challenges, improved storage stability for EPF has been achieved through successful encapsulation in a number of studies. Liu and Liu [98] experimented with various materials for coating *M. anisopliae* conidia, finding that a formulation using hydroxypropyl methyl cellulose (HPMC; 0.03% *v*/*v*) resulted in 78% encapsulation efficiency and maintained an 80% conidial germination rate after six months of storage at 4 °C, markedly better than the 50% rate for unformulated conidia under identical conditions. In a related study focusing on *B. bassiana*, low-temperature spray drying proved more effective for capsule production than conventional methods. Conidia encapsulated with a blend of dextrin (10% *w*/*v*), skim milk (2.5% *w*/*v*), and polyvinylpyrrolidone (PVP) K90 (10% *w*/*v*) retained an 80% germination rate after six months at 4 °C, compared to 60% for the unformulated control [99].

Beyond enhancing stability, encapsulation can also contribute to increased efficacy against target pests. A notable recent example involved an encapsulated *B. bassiana* formulation tested against the two-spotted spider mite, *Tetranychus urticae* Koch, on jack bean (*Canavalia ensiformis* (L.) de Candolle) plants. Both greenhouse and field experiments revealed that the encapsulated formulation exhibited significantly greater effectiveness compared to an unformulated commercial counterpart, inducing up to 6.6 times higher mortality rates [100].

Among the several types of polymers that can be used to construct the capsules, biopolymers represent a suitable option for the development of environmentally safe formulations. By definition, biopolymers are natural or synthetic polymers, derived from renewable or non-renewable sources, that are degradable by living organisms [101], breaking down into smaller molecules, such as water or carbon dioxide [102]. The most common biopolymers used for the construction of EPF capsules are alginate, chitosan, and starch [94], but many others can be used, such as gelatin, polylactic acid, polyglycolic acid, cellulose, gellan gum, xanthan gum, gum Arabic, pectin, and carrageenan [93,96,103].

Fungal formulations using biopolymers have been shown to retain fungal viability for longer times and to increase insect mortality. *Metarhizium anisopliae* formulated in Na-alginate beads caused higher mortality in three species of white grubs (*Holotrichia serrata* (Fabricius), *Lepidiota mansueta* Burmeister, and *Adoretus* sp.) when applied in concentrations of 500 mg and 1000 mg (2.1 × 10^8^ CFU/g), compared to an unformulated spore suspension; moreover, even after 10 months at room temperature, beads dried using freeze-treatment remained viable [104]. Packed beads of Ca-alginate containing 20% corn starch and *M. brunneum*, kept at 5 °C, retained viability without decreasing it during a period of six months [105]. The viability of *B. bassiana* encapsulated in Na-alginate remained stable and did not decrease during storage for a period of 12 months [96] or after exposure to UV radiation for 48 h [106]. In a study with *B. bassiana*, Felizatti et al. [97] compared six biopolymers as encapsulation agents (soy oil, corn starch, cellulose, lignin, alginate and humic acid), and showed that, in general, formulations had an increase in thermal stability and UV tolerance, and resulted in higher mortality of *Spodoptera cosmioides* (Walker), compared to unformulated material.

Despite the advantages, the use of biopolymers to encapsulate EPF requires more research to make this type of formulation more competitive in the market. During development of the beads, many properties of the biopolymers can impact the formulation, such as solubility, polarity, viscosity, and gelation mechanism [107]. Besides that, the inundative delivery method makes it less cost-efficient than synthetic insecticides [108], and the higher production cost and shorter shelf life compared to traditional formulations or synthetic polymer-based formulations [94,109] make them less attractive.

### 3.2. Encapsulation in Attract-and-Kill Strategies

The Attract-and-Kill (AK) strategy, also commonly referred to as Lure-and-Kill (LK), involves the targeted application of a semiochemical attractant combined with an insecticidal agent within a defined area. The semiochemical serves to draw target pests towards the point source, where they encounter and acquire a lethal dose of the insecticide. The principles, advantages, limitations, and factors influencing the success of AK programs have been comprehensively reviewed by El-Sayed et al. [110]. This pest management tactic has been employed for decades, utilizing various insecticidal components, including synthetic chemical insecticides [111,112,113,114], plant-derived volatiles [115,116] and pathogenic microorganisms like bacteria [117] and EPF [118,119,120,121,122,123]. AK approaches are considered particularly advantageous against soil-dwelling, herbivorous insects that rely on chemical cues, such as root exudates, for host plant location [124,125].

In recent years, a novel adaptation of AK strategies employing EPF has garnered significant attention for controlling subterranean insect pests like wireworms (*Agriotes* spp.) and the western corn rootworm (*Diabrotica virgifera* Leconte). This approach utilizes calcium- or sodium-alginate-based beads to co-encapsulate baker’s yeast [*Saccharomyces cerevisiae* (Desm.) Meyen], acting as a carbon dioxide (CO_2_) source, along with a suitable nutrient source and the EPF. Upon application to moist soil, the dried beads rehydrate, activating both microorganisms: the yeast metabolizes the nutrient to produce CO_2_, which functions as the attractant, while the EPF proliferates within and emerges from the bead as mycelia, subsequently sporulating on the surface to act as the killing agent [126,127].

Several challenges must be addressed for this system to be effective. Continuous CO_2_ emission is required throughout the potentially extended hatching period of target insects, which can span several weeks [128]. Furthermore, conventional encapsulation techniques are often inefficient at retaining low molecular weight nutrients (e.g., simple sugars) within the bead matrix [93]. While higher molecular weight polysaccharides like starch can be effectively incorporated, *S. cerevisiae* lacks the necessary amylase enzymes to directly utilize starch [129]. An attempt by Vemmer et al. [130] sought to resolve this by co-encapsulating *S. cerevisiae*, maize starch, and *B. bassiana*, leveraging the fungus’s amylase production. While this combination initially enhanced CO_2_ release compared to previous approaches [131,132] and transiently reduced larval presence near maize plants, it ultimately attracted more larvae after 24 h without causing infection, as the fungal sporulation lagged behind larval arrival. This highlighted the critical temporal mismatch and underscored the need to consider environmental factors, native microbial interactions, and potentially phagostimulants to retain insects near the beads longer [130]. Another consideration that may resolve this issue is earlier application of EPF: by seeding the field with EPF one-to-two seasons before the primary crop, EPF can establish in the field in abundance, before a second semiochemical-EPF co-formulation is applied to achieve maximal effect [133].

As an alternative strategy to co-encapsulation of all components, Brandl et al. [134] employed two distinct alginate bead types for wireworm control: one containing *S. cerevisiae* and starch (“Attract”), and another containing *M. brunneum* and inactivated yeast as an energy source (“Kill”). Co-application of these bead types successfully increased fungal abundance in the soil and reduced damage to potato crops by 37–75%, compared to the untreated control. Addressing the critical time lag between pest attraction and fungal sporulation, Hermann et al. [135] applied polyvinyl alcohol film coatings containing *M. brunneum* blastospores onto AK beads. These coatings acted as an immediate killing agent against *Tenebrio molitor* L. larvae, effectively bridging the temporal gap until the encapsulated fungus sporulated, significantly reducing lethal times (LT_50_ and LT_75_ decreased by 40%) and enhancing overall fungal sporulation from the beads.

Optimizing the physical properties of the beads, particularly water absorbency, which directly impacts microbial activity, is also crucial. Incorporating formalin-casein into Na-alginate-based “Kill” (corn starch + *M. brunneum*) or “AK” beads (Kill + *S. cerevisiae*) was shown to improve bead rehydration rates, enhance CO_2_ production, and boost conidia formation by *M. brunneum* [136]. Further potential for improving alginate gel characteristics exists through established methods like chemical crosslinking [137] or enzymatic modification [138], offering avenues for enhancing AK bead performance.

Regarding scalability, investigations into the mass production of EPF-based AK beads remain limited. Humbert et al. [126] reported the successful technical-scale production of separate “Attract” and “Kill” beads using jet cutting technology coupled with fluidized-bed drying, achieving throughputs of 5.15 kg/h and 4.13 kg/h, respectively, with a likely potential for upscaling via multi-nozzle systems.

In summary, the application of alginate-encapsulated EPF within AK strategies presents a promising, biologically based alternative to synthetic polymers and chemical insecticides for managing soil-dwelling pests. However, research is still required to fully elucidate the complex interactions between the co-encapsulated microorganisms, optimize bead attractiveness and persistence, validate efficacy under diverse field conditions, and develop cost-effective large-scale production methods necessary for market competitiveness.

### 3.3. Pickering Emulsions

Pickering emulsion technology, a microencapsulation method first documented over a century ago [139,140], has experienced a resurgence of scientific interest since the early 2000s [141]. Distinct from conventional emulsions reliant on molecular surfactants for stabilization, Pickering emulsions employ solid nanoparticles (referred to as Pickering stabilizers) that spontaneously self-assemble at the oil-water interface, acting as a physical barrier and providing stability to the emulsion against coalescence [142,143]. Consequently, the characteristics and surface properties of the nanoparticles are pivotal factors determining emulsion formation and stability. Extensive reviews, such as those by Yang et al. [144] and de Carvalho-Guimarães et al. [145], provide detailed descriptions of various nanoparticle types used for Pickering stabilization and discuss their application in diverse fields, including biomedicine.

While Pickering emulsions have been extensively utilized in the pharmaceutical and food industries for many years [146,147,148,149], their application for formulating EPF is still in a comparatively early phase of investigation. Pickering emulsions can be formulated as either oil-in-water (O/W) or water-in-oil (W/O) systems, depending primarily on the wettability characteristics of the stabilizing nanoparticles. However, W/O emulsions are generally deemed unsuitable for EPF conidia due to the inherent hydrophobicity of the conidia and the potential for premature germination triggered by contact with the continuous aqueous phase.

Studies have demonstrated the feasibility and potential benefits of this approach for EPF. Yaakov et al. [150] achieved successful single-cell encapsulation of *M. brunneum* conidia utilizing amine-functionalized silica nanoparticles as stabilizers. Subsequent application of this Pickering emulsion formulation to *Ricinus communis* L. leaves showed improved conidial distribution across the leaf surface and resulted in significantly higher mortality rates for *Spodoptera littoralis* (Boisduval) larvae feeding on these leaves, compared to control treatments involving an aqueous fungal suspension, the emulsion base without conidia, or distilled water. Corroborating these findings within the same experimental system, Birnbaum et al. [151] also showed enhanced conidial acquisition by larvae, reduced larval growth (body length), and decreased herbivory damage on leaves treated with *M. brunneum* Pickering emulsion. It was noted, however, that some effects on larval size and leaf damage were also evident with the conidia-free emulsion, suggesting the paraffinic oil carrier possessed intrinsic insecticidal activity and potentially acted synergistically with the EPF to increase overall lethality [151].

Enhanced protection against environmental stressors, particularly UV radiation, represents another significant advantage demonstrated for EPF Pickering emulsions. Feldbaum et al. [152] reported that a Pickering emulsion formulation of *M. brunneum* conidia stabilized with amine-functionalized titania nanoparticles displayed remarkable UV tolerance, maintaining germination rates above 80% after 3 h of exposure (42.6–363.4 Watt/m^2^ flux); this was statistically indistinguishable from unexposed controls and significantly superior to conidia suspended in an aqueous surfactant solution. Similarly, Shin et al. [153] showed that *B. bassiana* conidia encapsulated within a Pickering emulsion stabilized by cellulose nanofibrils could withstand 4 h of UV-C irradiation. Furthermore, encapsulated conidia remained highly virulent even after one hour of exposure to UV-C, resulting in *T. molitor* larval survival rates of 23.33% which is comparable to those achieved with an unexposed aqueous conidial suspension (16.67% survival rate).

Collectively, these findings highlight the considerable potential of Pickering emulsions as advanced delivery systems for EPF, offering prospects for enhanced efficacy and improved protection against environmental degradation. Nevertheless, substantial challenges remain before this technology can be considered a fully viable alternative to conventional pest control methods. Current limitations include often low encapsulation efficiencies, with reported yields reaching up to 44% [154]; and inadequate long-term stability, as evidenced in a study reporting conidial viability of only three weeks within an emulsion [150]. Furthermore, potential human health and environmental concerns associated with the use and accumulation of inorganic nanoparticles like silica and titania necessitate research into safer, biodegradable stabilizing materials [153]. Likewise, although paraffinic oils are generally regarded as safe, they can still pose ecotoxicity risks to non-target organisms such as aquatic invertebrates and soil fauna, depending on application rates and crop type [155]. Their moderate to high persistence in soil and strong adsorption to sediments may result in localized environmental effects under certain use conditions, while groundwater contamination is unlikely. Precise control over emulsion parameters, including nanoparticle concentration and the oil-to-water phase ratio, is also critical for achieving stable emulsions with appropriate droplet sizes that accommodate the fungal propagules. Addressing these challenges related to production yield, shelf life, safety, and formulation optimization through continued research is imperative for realizing the full potential of Pickering emulsions in mycoinsecticide development.

### 3.4. Comparative Assessment of EPF Formulation Approaches

Across the different EPF formulation types, commercial uptake and usage is dependent on a mixture of field performance, usability and cost. Traditional powder-based formulations such as WPs and DPs remain widely used due to their low cost, ease of handling, and compatibility with conventional spray equipment [27,34]. These advantages, combined with comparatively good storage stability for a biopesticide [42], explain their continued relevance despite the development of more advanced products. Nevertheless, powder formulations provide limited protection against environmental stressors and are largely associated with dust hazards for users, inconsistent coverage during application, and reduced rainfastness [41,43]. These weaknesses are particularly evident in foliar environments, where rapid conidial inactivation remains a major constraint.

Granular formulations occupy a distinct niche among EPF formulations. Their strengths include suitability for soil-dwelling pests and long-term persistence in soil, where they tend to be the most effective and persistent EPF formulation strategy [51,52,53,156]. Granules also present fewer handling risks than powders and provide more controlled, localized delivery. However, their efficacy strongly depends on adequate moisture availability, which governs the rehydration and germination of propagules. Furthermore, granules generally exhibit slower action than foliar sprays, and they are largely suitable only for subterranean sowing, with limited applicability to above-ground pests.

Liquid, oil-based formulations (e.g., OD, OF, UL) generally provide greater protection to fungal propagules in above-ground environments, improved adherence to plant surfaces, and increased virulence against target insects [74,76,78,80], making them highly suited to foliar pests where water-based or powder formulations would be less effective and more cost-prohibitive. Nevertheless, oil-based products also present challenges, including higher costs and, in the cases of many OF and UL formulations, dependence on specialized ULV equipment, which restricts their wider uptake and use.

Encapsulation technologies, including biopolymer-based matrices, alginate beads, and Pickering emulsions, represent promising but early-emergent strategies. These systems can enhance environmental protection, provide controlled release, and, in the case of attract-and-kill systems, the ability to combine attractants with infective propagules [91,93,126]. Improved storage stability and increased tolerance to UV or heat stress have been reported for several encapsulation formulations [97,99,106], as well as higher efficacy against pests [100]. Despite these advantages, important limitations remain, including low encapsulation yields in some systems [154], high production costs for biopolymers [94,109], and limited evidence for multi-season field persistence. For Pickering emulsions, the nanoparticle stabilizers raise questions regarding their environmental fate and regulatory acceptance [153].

Knowledge gaps persist across all formulation classes; closing these gaps and addressing the core concerns mentioned above is going to be a key determinant in the long-term uptake of EPF products over other control agents. For powder-based formulations, key uncertainties relate to the extent to which new additives can reliably reduce field variability. For oil-based systems, questions remain concerning the cost-effectiveness of large-scale deployment in lower-income agricultural systems. For granular formulations, relatively few studies have assessed interactions with soil physicochemical properties, indigenous microbial communities, or their potential for endophytic colonization. Encapsulation technologies exhibit the most pronounced gaps: encapsulation efficiency, environmental fate of encapsulating materials, and scalability of manufacturing processes require further investigation.

The formulation types discussed occupy distinct functional niches, each shaped by their physicochemical properties and intended applications. Continued research across these formulation classes, aiming at improving stability, reducing production costs, and clarifying interactions with plants, soil, and the surrounding microbiome, will be essential for expanding the amount of effective EPF-based mycoinsecticides.

## 4. Entomopathogenic Fungi as Endophytes: Formulation and Inoculation Strategies

While the preceding sections focus on classical and innovative formulations designed to maximize the survival, dissemination, and efficacy of EPF, it is important to acknowledge their broader ecological versatility. Beyond their role as insect pathogens, many EPF species can interact with plants as endophytes, colonizing internal plant tissues without causing disease symptoms [157,158,159]. Through these associations, EPF can provide additional benefits to plants, including enhanced growth and productivity [160,161,162,163], improved tolerance to abiotic stresses, such as drought and salinity [164,165], and protection against plant pathogens [166,167] and herbivore pests [168,169,170,171]. Harnessing this dual lifestyle expands the role of EPF in IPM programs by integrating insect suppression with plant protection and growth promotion.

Because endophytic establishment depends on propagule viability, survival on plant surfaces, adhesion, and timing of germination, the formulation used to deliver the EPF has a direct influence on whether endophytism is achieved and how extensively fungi colonize plant tissues. Different formulation types influence colonization by protecting propagules from abiotic stress, modifying nutrient availability, and determining their placement on seeds, leaves, or roots. As a result, formulation plays a key role in successful endophytic establishment, while the inoculation strategy further shapes the persistence and distribution of fungal colonization within host plants.

Several methods have been tested to establish EPF as endophytes, with varying degrees of success depending on the plant species, environmental conditions, and the fungal species and strain; notably, significant differences in metabolite repertoires have been reported among conspecific isolates [172], highlighting the need to investigate each isolate individually to account for these differences.

Seed treatments encompass techniques in which the a.i. is inoculated or applied to the seed prior to sowing. Upon germination, the released EPF can colonize root tissues and establish in the rhizosphere. The simplest approach is seed dressing, which involves immersing seeds in a fungal suspension prior to sowing [173], and has successfully achieved endophytic colonization in beans, corn, and cotton, providing protection against insect pests and promoting plant growth [174,175,176,177]. A more advanced approach, seed coating, involves applying polymers or adhesives to the seed surface, modifying its physical properties while serving as a carrier for the a.i. (EPF propagules, fungicides, or insecticides), helping to prevent loss of the a.i. during coating and sowing, as well as protecting the propagules [178,179,180,181]. In oilseed rape, coating seeds (pre-coated with insecticide and fungicide) with *B. bassiana* demonstrated that the fungus can tolerate certain fungicide concentrations, suggesting compatibility with common chemical seed treatments. Furthermore, inclusion of 2% gelatine and 1–4% baker’s yeast as nutrients in the coating enhanced fungal growth on the seed [182].

Foliar application, commonly in the form of WPs or ODs, represents another approach to establishing endophytic associations. Following spray application, conidia may germinate and enter the plant through penetration of the epidermal cell wall or through natural openings such as stomata [183]. Several studies have demonstrated that spraying leaves with suspensions of EPF, such as *B. bassiana*, *Beauveria brongniartii* (Sacc.) Petch or *M. brunneum*, can result in systemic presence of fungi within stems and leaves, and occasionally within roots [184,185,186]. Formulation composition can greatly affect the survival and capacity of conidia to germinate on leaf surfaces and invade tissues, as components such as UV protectants, oils, or humectants influence the persistence of fungal propagules on leaf surfaces, and consequently the window during which they may invade plant tissues. Lohse et al. [182] reported that a liquid formulation of *B. bassiana* containing UV protectants (1% titanium dioxide, 5% sugar beet molasses, or 0.1% Triton X-114) increased propagule survival and resulted in higher detection levels of endophytic colonization compared to a water-based control. These results highlight that classical foliar spray formulations, generally developed for pest suppression, can also be optimized to enhance endophytic establishment by prolonging propagule viability on the phylloplane.

Soil applications of EPF represent a third inoculation approach. Once introduced into the soil matrix, fungal propagules may colonize roots as endophytes or persist in the rhizosphere. Drenching the soil with fungal suspensions, usually applied at the base of the plant, has led to successful endophytic colonization of plants such as cassava, wheat, and maize [187,188,189]. Granules containing microsclerotia are especially promising, as they are more tolerant to desiccation than other propagules, release infective conidia following rehydration, and can directly interact with the developing root systems [51,53]. Soil applications combine pest suppression, especially against soil-dwelling insects, with the added potential for endophytic establishment, thereby extending protection to both roots and aerial plant parts. Although GR formulations are mainly used for insect control, and not many studies have addressed them as carriers of EPF to achieve endophytism [190], the available evidence indicates that the slow-release and hydration properties of granules can create favorable conditions for root colonization. For instance, encapsulation of *M. brunneum* mycelium in 2% Ca-alginate beads containing 20% corn starch enhanced fungal colonization in tomato plants by 3.8–7.0-fold compared to unformulated mycelium [191]. Similarly, *M. brunneum* mycelium encapsulated in 2% Ca-amidated pectin beads containing 1 U/g cellulase increased endophytism by over 60% compared to beads without the supplements [192], demonstrating how encapsulation materials and additives can modulate release, nutrient availability, and ultimately endophytic colonization.

Overall, the establishment of EPF as endophytes highlights the multifunctional potential of these fungi beyond direct insect pathogenicity. Successful endophytic colonization depends not only on the fungal strain and plant species but also on the choice of formulation and application method, which influence propagule survival, adhesion, germination, and release at the plant surface or root-soil interface. Although most classical formulations (e.g., WPs, GRs) were originally designed for pest suppression, their adaptation to seed coating, foliar spraying, or soil inoculation offers opportunities for innovation, such as incorporating components that enhance adhesion to seeds or leaves to promote endophytism and achieve synergistic benefits for crop protection, insect control, and plant health within integrated pest management programs.

## 5. Regulation of Mycoinsecticides

The emergence of bioinsecticides as eco-friendly alternatives to chemical insecticides has led governments around the world to create new regulations or to adapt existing ones to deal with this new share of products.

In the USA, the Environmental Protection Agency (EPA) regulates bioinsecticides under the Federal Insecticide, Fungicide, and Rodenticide Act (FIFRA) and the Federal Food, Drug, and Cosmetic Act (FFDCA). The EPA’s Biopesticides and Pollution Prevention Division (BPPD), established in 1994, handles plant protective product (PPP) registration. This process typically requires less extensive data, involves lower fees, and offers expedited timelines for bioinsecticides compared to conventional chemicals, due to their generally lower risk profile [193]. Despite these advantages, mycoinsecticide registration remains low. As of July 2025, only eight end-use mycoinsecticide products were listed as “active” in the EPA’s Active Pesticide Product Registration Informational Listing (APPRIL) database, within the “insecticide, microbial pesticide” category (out of 92 products) [194]. Maximum Residue Limits (MRLs) are determined by the EPA, according to the Code of Federal Regulations. Fungal strains can be granted an exemption from tolerance (i.e., no MRL established) if their presence in or on raw agricultural commodities is deemed non-hazardous to public health when used per good agricultural practices [195]. The EPA also regulates environmental safety through tiered assessments of impacts on non-target organisms. Human health toxicology follows mandatory Tier I tests (e.g., acute oral/dermal toxicity, inhalation, eye irritation), with Tier II/III tests required if adverse effects appear. Effects on non-target organisms and environmental fate also follow a tiered system, with Tier I covering toxicity/pathogenicity on birds, mammals, fish, aquatic invertebrates, plants, non-target insects, and honey bees; advanced Tier II-IV tests may be required if adverse effects are detected [196].

In Brazil, bioinsecticide registration requires joint approval from the Ministry of Agriculture, Livestock and Food Supply (MAPA), the Brazilian Institute of Environment and Renewable Natural Resources (IBAMA), and the Brazilian Health Regulatory Agency (ANVISA), with MAPA issuing the registration certificate. A 2011 Joint Normative Instruction streamlined and prioritized the registration of products suitable for organic agriculture [197], significantly boosting biopesticide numbers: 50 new products were registered from 2011 to 2016, surpassing the previous total of 41 [198]. The launch of the National Bioinputs Program [199] further accelerated the bioinsecticide market expansion by promoting biobased products derived from Brazilian biodiversity. As of July 2025, Brazil had registered 259 EPF-based microbial insecticide/acaricide products [200]. The ANVISA evaluates pesticide safety and assesses MRLs, but does not establish MRLs for mycoinsecticides [201]. Environmental risk assessment involves all three agencies. The ANVISA mandates a toxicological dossier with mammalian studies, while the IBAMA requires an ecotoxicological dossier, including studies on non-target species (earthworms, bees, birds, fish, microcrustaceans, algae) and on carbon and nitrogen transformation, with soil microorganisms involved in nutrient cycling processes [201].

In the EU, Regulation (EC) No 1107/2009 governs PPPs [202]. Approval is a two-step process: first, active ingredients require EU-level approval following risk assessment by a rapporteur Member State (RMS) and peer review by the European Food Safety Authority (EFSA) with other Member States (MS). Subsequently, the PPP can be submitted for approval at the MS level, coordinated by a zonal RMS. Residues are regulated under Regulation (EC) No 396/2005 [203], with the EFSA establishing MRLs based on a risk assessment. Low-risk active substances can be listed in Annex IV of this regulation, exempting them from MRLs. As of July 2025, 12 EPF isolates are approved as active substances, with 8 listed in Annex IV [204]. Environmental safety and non-target organism effects are detailed in Regulation (EC) No 1107/2009 (as amended by Regulation (EU) 2022/1438) [202], Regulation (EU) No 283/2013 (as amended by Regulation (EU) 2022/1439) [205], Regulation (EU) No 284/2013 (as amended by Regulation (EU) 2022/1440) [206], and Regulation (EU) No 546/2011 (as amended by Regulation (EU) 2022/1441) [207]. These documents outline the data requirements for the approval of microorganisms as active substances. The evaluation covers human health, metabolite fate, and comprehensive ecotoxicological studies on various organisms (terrestrial vertebrates, fish, aquatic invertebrates, algae, bees, non-target arthropods, soil organisms, and non-target plants). Additional studies may be required if adverse effects are found [205,206,207].

In China, pesticide registration is regulated by the Institute for the Control of Agrochemicals (ICAMA), under the Ministry of Agriculture and Rural Affairs (MARA). The legislation concerning the requirements for registration was updated in 2017 [208], and in the same year, the Data Requirements for Pesticide Registration were issued [209], specifying the requirements for active substances and formulated products across all pesticide classes, including microbial agents. The issuance of this document simplified registration testing procedures, reduced registration costs, and shortened approval timelines for biological products. In 2020, new policies were further introduced to prioritize and accelerate the evaluation of biopesticides and other low-risk pesticide products [210]. As of November 2025, a search on the ICAMA Data Center returned 35 products registered under *Beauveria* (all *B. bassiana*), 19 registered under *Metarhizium* (all *M. anisopliae*), 13 under *Paecilomyces* (all *Paecilomyces lilacinum*, currently *Purpureocillium lilacinum*), and 4 under *Cordyceps* [all *Cordyceps javanica* (Frieder. & Bally) Kepler, B. Shrestha & Spatafora]. No entries are recorded for *Akanthomyces* and the former name *Lecanicillium*; under the former name *Verticillium*, five products are found, all based on a strain used to control nematodes, but not insects. It is worth mentioning that the database is not searchable by category and requires prior knowledge of the active ingredient, making it impractical to retrieve a complete list of mycoinsecticides. Microbial biopesticides do not have MRLs, but residue tests may be required in case of toxicological significance. Environmental impact assessment requires toxicology tests on humans and impacts on birds, fish, bees, silkworms and planktonic crustaceans [209].

A comparative overview of the main regulatory features for microbial pesticides in these four regions is presented in Table 3. These regulatory contrasts have broader implications for innovation and commercialization within the mycoinsecticide sector. In the USA, microbial pesticide registration is supported by a dedicated biopesticides division and reduced data requirements. Brazil has adopted an accessible, biologics-friendly framework that has facilitated the rapid market expansion, despite the involvement of multiple agencies. The EU remains the most demanding jurisdiction, with stringent data requirements that limit the number of new microbial active substances entering the market. China has established a centralized regulatory system for all types of pesticides, combining simplified data requirements for biopesticides with policy instruments that prioritize the evaluation of low-risk products, promoting the development and registration of biological agents. Collectively, these regulatory differences shape not only the diversity of available mycoinsecticides but also the types of formulations and innovations that are commercially feasible in each region.

## 6. Market Trends and Expansion of Fungal Biopesticides

As a result of advances in formulation technology and EPF efficacy, alongside favorable changes in regulatory processes, the biopesticides market is, at present, experiencing faster growth compared to traditional chemical insecticides. The compound annual growth rate (CAGR) is projected to be 11–16% during the 2025–2035 period [211,212,213]. The estimated value of the biopesticides market varies according to the market research source, but the most common estimate is that it exceeded USD 8 billion in 2024, and depending on the estimated CAGR, the value is expected to slightly exceed USD 20 billion or nearly reach USD 30 billion by the early 2030s [211,212].

Within the biopesticides sector, fungi represent a significant share of the available products. However, reporting on this topic is generally outdated, with many recent publications still relying on incomplete, missing, or obsolete citations. For instance, several peer-reviewed papers published as recently as 2024 have stated that fungal biopesticides account for 10% of the global biopesticides market, citing earlier sources. Our review traced this information back from Thakore [214], an estimate that is nearly twenty years old. According to recent market reports, bioinsecticides currently account for approximately 37–40% of the biopesticides market [215,216]. Within the bioinsecticide sector, *B. bassiana* represents 20% of the market share, *M. anisopliae* roughly 15% and *A. lecanii* (including products with former *Verticillium lecanii* and *Lecanicillium lecanii* classifications) around 10% [217]. Based on these estimates, mycoinsecticides likely represent approximately 16.7–18% of the biopesticides market, a value notably higher than the outdated 10% value that continues to be widely cited.

Chandler et al. [218] reported that, in 2010, the EU had 34 registered microbial biopesticides, while the USA had 102. As of July 2025, the number of approved microbial biopesticides in the EU has increased to 73, with an additional 25 pending approval. In the USA, 155 microbial products are currently registered. Brazil, another major biopesticide market, has 612 currently registered microbial biopesticides. Table 4 presents the proportion of fungal-based products (mycoinsecticides and mycopesticides) relative to the total number of microbial products in these three major markets. Notably, not only has the total number of registered mycoinsecticides increased, but the proportion of mycoinsecticides and mycopesticides relative to the total biopesticide and bioinsecticide markets has significantly changed over the last two decades. For the following market analysis, a selection of the world’s major markets was sought out; however, only those jurisdictions for which complete, searchable, and product-level regulatory databases are publicly accessible were able to be included in the quantitative market analysis, primarily to ensure only primary data sources were used in a consistent manner. Our current analysis of EU, USA, and Brazilian product registration databases reveals that mycopesticides represent approximately 51.7% of the registered microbial biopesticides for all regions combined (with a regional variation of 13.6–60.8%), and mycoinsecticides account for an average of 59.59% of the registered microbial insecticides on these markets (ranging between 8.6 and 69.6% across regions). Although these figures represent only a subset of registered products across the world, they represent three of the largest biopesticide markets and provide an updated aspect of the relative changes and expected product ratios that would be relevant in today’s markets. Despite the relatively modest number of fungal-based products, fungi now appear to be the principal contributors to new microbial biopesticide developments, especially within the insecticide segment.

The differences shown in Table 4 illustrate how regulatory environments, domestic industry structure, and patterns of product adoption shape the composition of microbial markets. Brazil stands out with both the highest absolute number of registered microbial pesticides and the largest proportional share of fungal-based products. This pattern is consistent with the country’s recent policy incentives promoting microbial biopesticides and its substantial domestic manufacturing capacity, which together facilitate rapid product diversification. In the EU, stringent regulatory requirements continue to limit the total number of approved microbial products; however, among those that do reach the market, fungi constitute a relatively high proportion, reflecting a regulatory preference for well-characterized agents and the strong role of EPF within European biological control programs. In contrast, the USA, despite having a dedicated biopesticides division with comparatively streamlined processes, shows a markedly lower proportional representation of fungal products. This likely reflects a long-standing dominance of bacterial and viral microbials in the US market, combined with historically lower user uptake of mycoinsecticides.

Persistent constraints associated with fungal products, including higher production costs, slower speed of kill compared to chemical insecticides, and sensitivity to storage and environmental conditions, continue to influence commercial trajectories across all three regions. These constraints can temper adoption even in expanding markets, helping explain why rapid global growth in biopesticides does not always translate into proportional increases in fungal products. Nevertheless, ongoing technological advances in formulation and increasing regulatory and market support for biological control suggest that EPF-based products are positioned to contribute an increasingly significant share of future biopesticide growth.

Providing reliable and up-to-date information is of paramount importance to researchers, the biopesticides industry (whether small and medium-sized enterprises or large multinational companies), and stakeholders such as funding agencies. To support accurate assessments of market size and trends, continued research is necessary to generate robust, verifiable data. Regularly updated and well-referenced information will help guide and advance both scientific progress and industrial development within the biopesticides sector.

## 7. Challenges in Development and Implementation

EPF possess inherent characteristics that position them as promising candidates for biological control programs. Many species exhibit a broad host range; for example, *B. bassiana* reportedly infects over 700 insect species across 15 orders [3], and *M. anisopliae* infects over 200 species from 7 orders [2]. They typically also demonstrate some degree of specificity, presenting minimal to negligible risk to non-target organisms, including vertebrates and beneficial arthropods. Furthermore, their biology allows for integration into various biological control concepts, including classical, augmentative, and conservation strategies.

As we have described, EPF can be formulated using diverse technologies, enabling their effective application for insect control under field conditions. However, despite the significant progress achieved in formulation science, several persistent challenges continue to impede the broad-scale commercialization and adoption of novel EPF-based products.

A primary factor limiting the field performance of mycoinsecticides is their susceptibility to environmental degradation. Key abiotic factors, including solar radiation (particularly UV wavelengths) and temperature extremes, can significantly inhibit fungal growth or lead to complete inactivation of infective propagules [21,219,220,221]. Rainfall can physically dislodge or wash propagules from target surfaces [78], while inadequate humidity levels can prevent the essential step of conidial germination [222]. The cumulative impact of these factors can result in suboptimal pest control, often necessitating more frequent applications and thereby increasing overall treatment costs and efforts. Advanced formulations incorporating UV-protectant additives (e.g., specific pigments or sunscreens), oils, or employing encapsulation techniques (e.g., with nanoparticles or biopolymers) represent key strategies to shield fungal propagules from these environmental insults.

Reduced efficacy can also result from the loss of propagule viability during product storage and transportation. Mycoinsecticides may be exposed to temperatures ranging from 40 to 60 °C within transit vehicles, warehouses, or on-farm storage facilities, particularly in tropical and subtropical climates [223,224]. This thermal stress can be especially detrimental to less protected products, such as technical concentrates, which remain widely used in regions like Brazil [96,198]. Achieving adequate stability across these conditions through formulation is challenging, as optimal storage requirements and thermal tolerance can vary considerably depending on the fungal species, fungal isolate, and the type of propagule being formulated (e.g., conidia vs. blastospores vs. microsclerotia).

Economic considerations also pose significant hurdles. The production costs associated with mycoinsecticides often exceed those of conventional chemical insecticides, contributing to a higher final product price; for instance, a *B. bassiana*-based product was reported to be nearly twice as expensive as certain pyrethroid-based alternatives [108]. High registration costs and, as mentioned, potentially more frequent application requirements compared to some chemical insecticides can further impact overall cost-effectiveness. Additionally, scaling up the production of innovative formulations often requires substantial investment in specialized mass production technologies, which can add to the final cost burden. Compounding these economic factors are challenges related to market perception. The inherently slower speed of kill associated with fungal infection cycles, compared to the rapid knockdown effect of many synthetic neurotoxins, can lead end-users to perceive mycoinsecticides as less efficacious. Furthermore, inconsistent field results, sometimes arising from improper storage, handling, or application timing by users unfamiliar with biologicals, can undermine confidence in EPF-based products. Enhanced education and outreach efforts targeting growers, distributors, and agricultural consultants are crucial for promoting correct usage, overcoming user resistance, and improving the market positioning and acceptance of mycoinsecticides.

Finally, significant global variations in microbial bioinsecticide registration frameworks directly impact research investment, market access, and economic incentives. Countries like Brazil, China, India, and the USA, for example, implemented tailored microbial bioinsecticide regulations during the 2000s and 2010s, stimulating new product development and subsequent market expansion. Conversely, the EU’s regulatory approach for microbial control agents has traditionally mirrored that of chemical insecticides. This is widely considered a key factor limiting innovation and market growth within the EU, leading to slower adoption of new microbial products compared to other regions [225]. Although the EU introduced microorganism-specific regulatory amendments in 2022, a detailed analysis of the challenges that have hindered its registration process of microbial control agents is provided by Sundh and Eilenberg [226].

Taken together, these challenges differ considerably in their current impact on field deployment. Abiotic factors, especially UV radiation, high temperatures, and relative humidity, remain the factors with the greatest impact on field performance. In contrast, issues related to storage stability and propagule protection are already being mitigated through formulation advances such as UV protectants, oil-based carriers, encapsulation systems, and stress-tolerant propagules. Highlighting this distinction helps contextualize which barriers currently constrain large-scale deployment the most, and which are likely to be addressed in the near term through ongoing formulation improvements.

## 8. Conclusions

EPF have been the subject of investigation as potential insect control agents for well over a century. Formulation technology plays an indispensable role in translating this potential into practical applications, enabling EPF to function as effective and environmentally compatible biological control agents, thereby presenting viable alternatives or complements to synthetic chemical insecticides.

The successful development and widespread commercial adoption of high-quality, efficacious, user-friendly, and economically competitive mycoinsecticides are pivotal for catalyzing increased investment in both research and market infrastructure. Such advancements also foster the refinement of regulatory mechanisms and the motivation of greater confidence in these biological tools among all stakeholders within the agricultural sector, including growers, retailers, and consultants. Actualizing this transition, from EPF potential to robust mycoinsecticide products, largely depends on sophisticated formulations. This development process must carefully integrate a multitude of factors, encompassing not only the intrinsic biological characteristics of the microorganism (e.g., species/isolate choice, propagule type, virulence traits, environmental tolerance limits) but also the specifics of the application context, including the target environment, delivery methods employed, and the biology of the pest organism.

Formulation science has undeniably achieved significant progress over the years, leading to improvements in the shelf life, environmental stability, and field efficacy of various EPF-based products. Nevertheless, continued, dedicated research and strategic investment are essential to overcome persistent obstacles. These include challenges related to the cost-effective, large-scale mass production (especially concerning newer formulation types, as discussed in Section 3), ensuring consistent long-term storage stability across diverse climatic conditions, achieving reliable and predictable field performance, and streamlining the often complex regulatory pathways required for product registration. Concurrently, ongoing developments in biotechnology present opportunities to further enhance EPF performance, with genetically engineered strains exhibiting traits like augmented virulence or increased resilience to environmental stressors already being demonstrated in research settings [227,228,229,230]. The multifaceted debate surrounding the potential regulatory approval and market acceptance of such genetically modified mycoinsecticides, however, falls outside the defined scope of this review.

Lastly, it is imperative to situate mycoinsecticides and bioinsecticides generally, within their appropriate operational context. They are optimally deployed not as universal replacements for chemical insecticides, but rather as valuable components integrated within comprehensive IPM programs. Cultivating a deeper understanding of the specific roles mycoinsecticides can fulfill within IPM, including their compatibility and potential synergies with other control methods (biological, cultural, physical, and chemical), is fundamental to constructing more sustainable and resilient pest management systems that strategically reduce reliance on broad-spectrum chemicals while maximizing the contributions of biological control agents like EPF.

## Figures and Tables

**Table 1:** Examples of current commercial formulations of mycoinsecticides.

Fungal Species	Commercial Name	Formulation ^a^	Target Pests	Company	Country
*Akanthomyces lecanii* ^b^	LECATECH WP	WP	Whiteflies	Dudutech	Kenya
	VERTI-SIN	SC	Aphids	Agrobionsa	Mexico
	*Verticillium lecanii* 1.15% WP	WP	Mealybugs	Peptech Biosciences Ltd.	India
*Beauveria* *bassiana*	*Beauveria bassiana* ZJU435	OD	Whiteflies	Julixin Bioengineering	China
	BotaniGard ES	OD ^e^	Whiteflies, aphids, thrips, psyllids, weevils, leafhoppers, grasshoppers, mealybugs, foliage-feeding Lepidoptera, scarab beetles	Certis Biologicals	USA
	Botanigard 22WP	WP			
	Boveril Evo	WP	Coffee berry borer, whitefly, sugarcane weevil, two-spotted spider mite, brown stink bug	Koppert do Brasil	Brazil
	Mycotrol ESO	OD ^e^	Whiteflies, thrips, aphids, psyllids, weevils, mealybugs, leafhoppers, scarab beetles, Orthoptera	Certis Biologicals	USA
	Mycotrol WPO	WP			
	Naturalis	OD	Whiteflies, mites, thrips, tephritid fruit flies, wireworms	Biogard	France, Greece, Italy, Spain
*Beauveria brongniartii*	Melocont GR	GR	Larvae of *Melolontha* spp.	Agroline	Switzerland
*Cordyceps* *fumosorosea* ^c^	Isarid	WP	Whiteflies, aphids, thrips, psyllids, mealybugs, leaf hoppers, mites	Koppert Biological Systems	USA
	PAE-SIN	SC	Whiteflies	Agrobionsa	Mexico
	PAE-SIN WP	WP		Biobest	Belgium, Finland, France, Netherlands, Norway, Sweden
	PreFeRal	WG			
*Cordyceps* *javanica*	Lalguard C99WP	WP	Whiteflies, maize leafhoppers	Lallemand Plant Care	Brazil
*Metarhizium acridum* ^d^	Green Guard SC Premium	OD ^f^	Locusts, grasshoppers	BASF	Australia
*Metarhizium* *anisopliae*	GranMet GR	GR	Larvae of summer chafer, European chafer and garden chafer	Agroline	Switzerland
	META-SIN	SC	Pepper weevils	Agrobionsa	Mexico
	META-SIN WP	WP			
	*Metarhizium* *anisopliae* CQMa421	OF/GR	Planthoppers, rice leafrollers, wireworms, chafers	Julixin Bioengineering	China
	Metarril WP E9	WP	Spittlebugs	Koppert do Brasil	Brazil
	Meta-Turbo SC	SC	Spittlebugs, fall armyworm, soybean looper, brown stink bug, tomato leafminer	Vittia	Brazil
*Metarhizium brunneum*	Lalguard M52 OD	OD	Thrips, whiteflies, mites, psyllids, weevils	Lallemand Plant Care	Austria, Belgium, Canada, France, Germany, Italy, Netherlands, UK, Ukraine, USA, Zambia

^a^ WP = wettable powder; SC = suspension concentrate; OD = oil dispersion; GR = granule; WG = water-dispersible granule; OF = oil miscible flowable concentrate. ^b^ Formerly identified as *Lecanicillium lecanii* or *Verticillium lecanii.* ^c^ Formerly identified as *Isaria fumosorosea* or *Paecilomyces fumosoroseus.* ^d^ Formerly identified as *Metarhizium anisopliae* var. *acridum.* ^e^ Emulsifiable suspension is a term commonly used to refer to oil dispersion (OD). Although the products are classified by the company as emulsifiable suspensions (ES), in the CropLife International code system, the abbreviation ES refers to emulsion for seed treatment; therefore, here we refer to the products as ODs. ^f^ Although the product is sold as a SC, its safety data sheet describes it as a viscous liquid with odor of essential oil, indicating that this is an OD rather than a SC. For further discussion on this, please refer to Section 2.3.2.

**Table 2:** Key advantages and disadvantages of the most traditional formulation types for entomopathogenic fungi.

Formulation Type	Advantages	Disadvantages
Wettable powder (WP)	• Easy to handle • Compatible with standard application equipment • Good shelf life	• Dust hazard • Risk of clogging equipment • Requires continuous agitation in the tank
Dustable powder (DP)	• Do not require water for application • Suitable for dry environments	• Dust hazard • Lower adherence to surfaces
Granule (GR)	• Targets soil-dwelling pests • Easy to handle and to apply • Extended residual effect in soil	• Slower action • Not suitable for foliar pests
Bait (ready for use) (RB)	• High specificity • Targets insects at the soil surface	• High specificity • Relies on pest attraction
Water-dispersible granule (WG)	• Dust-free • Shelf stable • Easy to store and to transport	• Lower efficacy compared to other formulations
Oil dispersion (OD)	• Enhanced protection against UV radiation and desiccation • Increased virulence against pests • Better adherence to plant surfaces	• Higher cost • Risk of phytotoxicity
Oil miscible flowable concentrate (OF)	• Compatible with several application methods	• High production cost • Limited availability
Ultra-low volume liquid (UL)	• Do not require water for application • Requires low volume of formulation • Suitable for aerial or ground equipment	• High equipment cost • Sensitive to wind drift

**Table 3:** Summary of regulatory characteristics for microbial pesticide registration in the USA, EU, and Brazil.

	USA	EU	Brazil	China
Main authorities	EPA’s Biopesticides and Pollution Prevention Division	EFSA and Member States	MAPA, ANVISA, and IBAMA	MARA’s ICAMA
Environmental safety	MRL may not be established depending on risk level; toxicology assessment on humans and non-target organisms follows a tiered system	Low-risk active ingredients can be exempted from MRL; requires data on human health, metabolite fate, and ecotoxicological studies on non-target organisms	MRLs not established for mycoinsecticides; toxicological dossier on mammals, ecotoxicological dossier on non-target species, and soil microorganisms	MRL not established, but may be required in case of toxicological significance; toxicology tests on humans and non-target organisms
Evaluation process	Centralized evaluation performed by the EPA	Multi-layered system involving EFSA and Member States	All three main authorities involved; registration certificate issued by MAPA	Centralized evaluation performed by ICAMA
Main bottlenecks	Occasional additional data requests for certain endpoints	Metabolite characterization, extensive dossier requirements	Possible delays due to inter-agency coordination	Centralized approval system with extensive dossier requirements
Impact on market entry	Supports registration of diverse microbial products	Limits number of new microbial active ingredients entering the market	Enables rapid growth in microbial product registrations	Supports and incentivizes registration of microbial products

**Table 4:** Proportion of fungal-based products among registered microbial products in Brazil, EU, and USA.

Region	Mycoinsecticides vs. Microbial Insecticides ^a^	Mycoinsecticides vs. Microbial Pesticides ^b^	Mycopesticides vs. Microbial Pesticides ^c^
Brazil	69.6%	42.3%	60.8%
European Union	48%	16.4%	56.2%
USA	8.6%	5.2%	13.6%

^a^ Microbial insecticides include bacteria-, fungi-, and virus-based products targeting insects or mites, as listed in each region’s databases (Brazil: Agrofit; EU: EU Pesticides Database; USA: APPRIL). ^b^ Microbial pesticides include bacteria, fungi, and virus-based insecticides, fungicides, nematicides, and/or bactericidal products. ^c^ Mycopesticides include fungal-based insecticides, fungicides, nematicides, and bactericidal products.

## Data Availability

No new data were created or analyzed in this study. Data sharing is not applicable to this article.
